# Influenza A(H1N1)pdm09 infection and viral load analysis in patients with different clinical presentations

**DOI:** 10.1590/0074-02760200009

**Published:** 2020-05-18

**Authors:** Vitória Rodrigues Guimarães Alves, Ana Helena Perosa, Luciano Kleber de Souza Luna, Jessica Santiago Cruz, Danielle Dias Conte, Nancy Bellei

**Affiliations:** 1 Universidade Federal de São Paulo, Departamento de Medicina, Disciplina de Infectologia, Laboratório de Virologia Clínica, São Paulo, SP, Brasil; 2 Hospital São Paulo, Laboratório Central, São Paulo, SP, Brasil

**Keywords:** influenza A(H1N1)pdm09 virus, viral load, rate of infection, asymptomatic.

## Abstract

**BACKGROUND:**

Influenza viral load (VL) can be a decisive factor in determining the antiviral efficacy in viral clearance.

**OBJECTIVE:**

This study aimed to evaluate the rate of infection and the role of influenza VL on the clinical spectrum of illnesses among different patient groups attended at a tertiary hospital in Brazil.

**METHODS:**

Samples were collected from patients presenting acute respiratory infection from 2009 to 2013. Overall, 2262 samples were analysed and distributed into three groups: (i) asymptomatic (AS); (ii) symptomatic outpatients (OP); and (iii) hospitalised patients (HP). VL (expressed in Log10 RNA copies/mL) was calculated through a quantitative real-time one-step reverse transcription-polymerase chain reaction (RT-PCR) assay aimed at the M gene, with human RNAseP target as internal control and normalising gene of threshold cycle values.

**FINDINGS:**

A total of 162 (7.16%) H1N1pdm09 positive samples were analysed. Patients aged from 0.08 to 77 years old [median ± standard deviation (SD): 12.5 ± 20.54]. Children with 5 to 11 years old presented the highest detection (p < 0.0001). AS patients had the lowest VL, with a significant difference when compared with symptomatic patients (p = 0.0003). A higher VL was observed within two days of disease onset. Ten patients (HP group) received antiviral treatment and were followed up and presented a mean initial VL of 6.64 ± 1.82. A complete viral clearance for 50% of these patients was reached after 12 days of treatment.

**MAIN CONCLUSIONS:**

It is important to evaluate AS patients as potential spreaders, as viral shedding was still present, even at lower VL. Our results suggest that patients with underlying diseases and severe clinical symptoms may be considered for prolonged viral treatment.

According to a recent study, up to 646,000 people die from respiratory diseases linked to season influenza each year.^1^ World Health Organization (WHO) data showed that between February and September 2019 infections with the Influenza A(H1N1)pdm09 (H1N1pdm09) virus were predominant in most countries in northern and eastern Europe, as well as northern Africa and Asia, and also in tropical countries of Asia and the Southern Cone of South America, when compared to others influenza type A viruses.^2^ In this scenario, this is still an important issue to be taken into account by health services around the globe.

Influenza infections can cause from mild to severe diseases or progress to a fatal outcome, particularly for high-risk groups, including children, pregnant women, elderly, immunocompromised patients, and those with chronic underlying medical conditions.^3^ ^,^ ^4^ People with influenza can express some or all of the following symptoms: fever, cough, sore throat, rhinorrhea, myalgia, headaches and fatigue. Vomiting and diarrhea were also observed, although being more common in children than adults.^5^ The disease can also present as asymptomatic, while still being able to implicate in transmission dynamics and control. For this matter, it is important to estimate the proportion of asymptomatic patients in influenza infections.^6^

Understanding the dynamics of H1N1pdm09 viral load (VL) can provide essential informations about the transmission and spread of the virus and also give insight into the infective agent-host interaction. It has been shown previously that a higher VL was associated with a more severe case of the disease in patients with ≥ 5 days of clinical symptoms, along with advanced age and comorbidities.^7^ ^,^ ^8^ ^,^ ^9^

VL dynamics can also be a decisive factor in determining the efficacy of an antiviral drug in the viral clearance of a patient. Especially now when new drugs have been recently approved by the US Food and Drug Administration (FDA) for treatment of influenza, such as baloxavir marboxil, a viral polymerase inhibitor.^10^

This study aimed to evaluate the rate of infection and investigate the pattern of VL in positive H1N1pdm09 samples in different patient groups attended at a tertiary hospital in Brazil.

## SUBJECTS AND METHODS

*Sample collection* - Respiratory samples were collected from patients presenting acute respiratory infection (ARI), including nasopharyngeal aspirates from children under 2 years of age at the enrollment visit. Nasal- and oropharyngeal swabs were collected from the other patients and placed into 2 mL of Ringer’s lactate sterile solution, according to instructions of the Brazilian Ministry of Health protocol for the management of Influenza A(H1N1) 2009 pandemic.^11^ Samples were collected from patients attended at primary care health service or specific units of the São Paulo Hospital, a tertiary hospital in the city of São Paulo, Brazil, from 2009 to 2013. Informed consent was obtained from all patients before collection according to physician demand and not on a regular basis. In this regard, sample collection from the different patient groups were not evenly distributed throughout the study period. After collection, samples were aliquoted and stored at -80ºC.

A total of 2262 samples were analysed and distributed into three different groups, regarding patient conditions: (i) asymptomatic (AS) (adults from the general community with absence of respiratory symptoms during 15 days before sample collection); (ii) symptomatic outpatients (OP) (children, adults, and healthcare workers from the general community); and (iii) hospitalised patients (HP), including children and adults with severe acute respiratory infection (SARI). H1N1pdm09 positive patients were also distributed into different standard aging groups according to WHO Epidemiological Surveillance Standards for Influenza, with modifications, as follows: < 2; 2-4; 5-11; 12-18; 19-58 and ≥ 59 years old.^12^ This study was conducted in compliance with institutional guidelines and approved by the Ethics Committee of São Paulo Federal University (CEP/UNIFESP n. 0700/2010 and 0542/2015).

*Laboratory methods* - RNA extraction of samples was carried out using the QIAmp Viral RNA Mini Kit (Qiagen, Hilden, Germany), according to the manufacturer’s instructions.

*VL calculation* - To calculate the VL of positive samples, a quantitative one-step real-time reverse transcription-polymerase chain reaction (RT-PCR) essay aimed at the M gene was performed, with the human RNAseP target as an internal control of sample quality and normalising factor of threshold cycle (Ct) values. VL was expressed in Log_10_ RNA copies/mL. Ct values were normalised according to the following equation: Ct = (sample Ct x RNaseP sample Ct)/(mean RNaseP Ct).^13^

*Data analysis* - Statistical analysis consisted of a chi-squared test for comparing categorical values, with a p < 0.05 being considered statistically significant. Results are presented as odds ratios with the respective 95% confidence intervals (CI). All statistical analyses were performed using GraphPad Prism v.6.01.

## RESULTS

A total of 162 H1N1pdm09 positive samples were analysed out of 2262 respiratory samples collected from asymptomatic (n = 15), hospitalised patients (n = 76) and symptomatic outpatients (n = 71).

Positive patients aged from 0.08 to 77 years old [median ± standard deviation (SD): 12.5 ± 20.54]. The age group of 5-11 years old presented the highest detection (p < 0.0001) (Table I).

**TABLE I:** H1N1pdm09 detection and viral load (VL) by age groups

Age groups (years)	Patients (n)	H1N1pdm09 (n %)	Odds ratio (95% CI)	VL^*a*^ (mean ± SD)
< 2	354	17 (4.8)	1	5.67 ± 1.71
2-4	183	15 (8.2)	1.71 (0.83-3.5)	6.82 ± 1.97
5-11	207	48 (23.19)	4.83 (2.71-8.62)*	6.75 ± 1.74
12-18	59	7 (11.86)	2.47 (0.98-6.21)*	7.29 ± 0.87
19-58	1276	63 (4.94)	1.03 (0.59-1.78)	6.17 ± 1.83
≥ 59	183	12 (6.56)	1.37 (0.64-2.92)	6.65 ± 2.35

*a*: Log_10_ RNA copies/mL; *: significant at p < 0.05; CI: confidence interval; SD: standard deviation.

Rates of infection by patient groups were of 3.14% (71/2262) for symptomatic outpatients, 3.36% (76/2262) for hospitalised patients and 0.66% (15/2262) for asymptomatic patients.

**Fig. 1:**
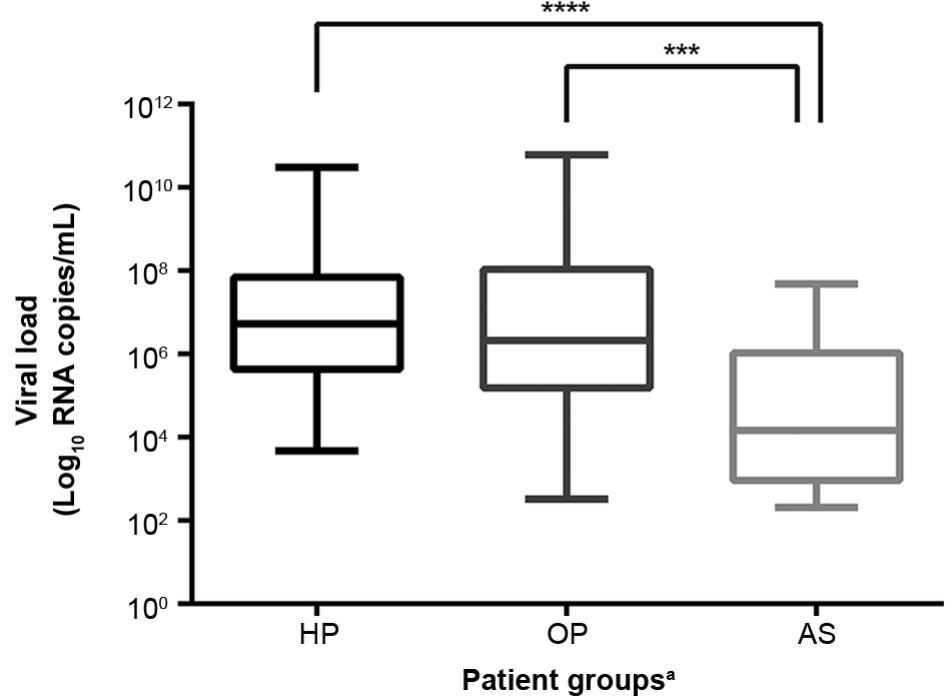
viral load (VL) distribution by patient groups. HP: hospitalised patients (children and adults); OP: symptomatic outpatients (children and adults); AS: asymptomatic patients (adults). Upper and lower bars indicate min and max VL values. Horizontal bars indicate median VL values. ***/**** significant at p < 0.05.

VL varied from 2.32 to 10.79 Log_10_ RNA copies/mL (mean ± SD: 6.43 ± 1.83). A significant difference was observed considering patient onset symptoms (asymptomatic vs. symptomatic - p = 0.0003) (Table II, Fig. 1).

**TABLE II:** Age and viral load (VL) (mean ± SD) by patient groups

Groups^*a*^	Patients (n %)	Age (years ± SD)	VL^*b*^ (mean ± SD range)
OP	60 (37)	5 ± 3.3	6.52 ± 1.91 (2.52-10.79)
	11 (6.8)	40 ± 16.5	6.18 ± 1.80 (3.36-9.64)
HP	21 (13)	5.6 ± 4	6.7 ± 1.5 (4.4-9.06)
	55 (34)	41 ± 16	6.89 ± 1.59 (3.67-10.49)
AS	15 (9)	37 ± 6.16	4.52 ± 1.76 (2.32-7.68)^*^

*a*: OP = symptomatic outpatients; HP = hospitalised patients; AS = asymptomatic patients; *b*: Log_10_ RNA copies/mL; *: significant at p < 0.05; SD: standard deviation.

VL (mean ± SD Log_10_ RNA copies/mL) obtained for children and adults was not associated to disease severity when considering the three groups analysed. The values varied as follows, respectively: 6.52 ± 1.91 and 6.18 ± 1.8 (OP); 6.89 ± 1.37 and 6.84 ± 1.55 (HP); 4.52 ± 1.76 (AS - adults). We also provide data analysis without Ct normalisation as supplementary material (Supplementary data **)**.

Days of onset symptoms until sample collection and respective VL were also analysed in the symptomatic outpatients group (Table III). A higher mean VL occured within 2 days of disease onset, with a statistically significant difference observed when comparing this value with the mean VL from patients with three to four days of onset symptoms (p = 0.016).

**TABLE III:** Days of onset symptom until sample collection and viral load (VL) of symptomatic outpatients (n = 71)

Onset symptom (days)	Patients (n)	VL^*a*^ (mean ± SD)	p
one - two	28	7.07 ± 1.54	0.073
three - four	33	5.97 ± 1.86	
five - eight	10	6.43 ± 2.48	

*a*: Log_10_ RNA copies/mL; SD: standard deviation.

**Fig. 2:**
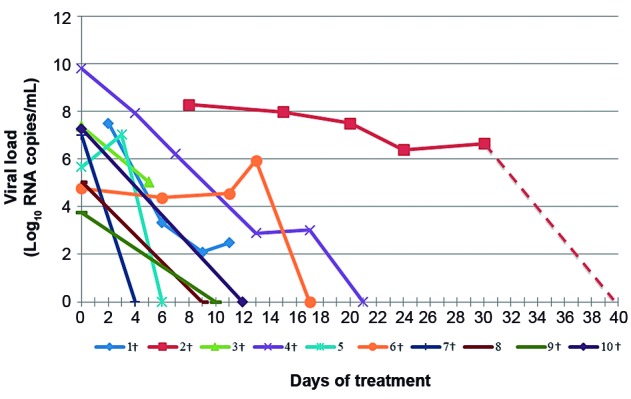
viral load (VL) of the 10 patients from the hospitalised patients group treated with oseltamivir. Each line indicates a different patient. Dotted line indicates last sample received on the 40th day of treatment from patient 2. †: presence of comorbidities. 1†: cardiomyopathy + atrial septal defect; 2†: asthma + smoker (under mechanical ventilation); 3†: tuberculosis; 4†: lymphoma (fatal outcome); 6†: lupus; 7†: neurologic disturbance; 9†: renal transplant; 10†: chronic liver disease.

Ten patients (13%) from the hospitalised patients group (n = 76), collected during the 2013 influenza season, were followed up for viral shedding analysis and serial samples were collected until clinical improvement and/or VL reduction. Measurements of VL according to days of oseltamivir treatment are shown in Fig. 2.

Followed up patients aged from 16 to 61 years (mean ± SD: 49 ± 13) with a mean initial VL of 6.64 ± 1.82 Log_10_ RNA copies/mL. The mean VL for patients with (8/10) and without comorbidities (2/10) was of 6.97 ± 1.91 and 5.36 ± 0.43 Log_10_ RNA copies/mL, respectively.

Only one (10%) patient presented a negative VL within five days of treatment with oseltamivir. H1N1pdm09 shedding was still detectable at the 10th day of treatment for 60% of the patients. Patient 2 VL was negative only after 40 days of drug treatment, VL values between the 30th and the 40th day could not be analysed due to absence of samples.

## DISCUSSION

Our results have shown that children with 5-11 years old presented the highest H1N1pdm09 detection (23.19%). A similar result was found in a previous study where children with ≥ 7 to < 13 years of age had a rate of infection of 44.1%.^14^ In contrast, an epidemiological study about influenza A in southern Brazil showed that adults with 21 to 30 years old were the most affected by the H1N1pdm09 virus, with a frequency of infection of 25.7%.^15^

Chung-Chen et al.^14^ also found that children with less than 13 years old presented a longer median duration of viral shedding than that observed for patients with more than 13 years of age (11 and seven days of shedding, respectively).

The asymptomatic patients group represented 9% of all H1N1pdm09 positive patients. A slightly higher proportion (12%) was found previously by Loeb et al.^16^ Asymptomatic patients had the lowest mean VL among all three groups (AS, OP and HP), which was expected since there were no previous reports to clinical signs or symptoms.^17^ Regardless, viral shedding was still present in these patients, even though with an asymptomatic form of the disease. Former studies have also reported viral shedding in asymptomatic patients, having a detectable shedding of an eight-day period with a mean duration of four days (SD of two days).^6^ ^,^ ^16^ These findings corroborate with the concern of the viral transmission potential that asymptomatic patients represent for H1N1pdm09 infections.

Considering the disease clinical spectrum, we found no significant difference regarding the mean VL of hospitalised and outpatients (6.84 ± 1.55 and 6.47 ± 1.88 Log_10_ RNA copies/mL, respectively) (p = 0.2). Contrarily, Lee et al.^9^ described a 1.4 Log_10_RNA copies/mL higher mean VL for hospitalised patients, when compared to outpatients (5.96 ± 1.19 and 4.51 ± 1.67, respectively) (p = 0.003).

When comparing VL values in different days of onset symptom, we found that a higher VL occured within two days of disease onset and tended to decrease with disease progression, which is in line with past studies.^8^ ^,^ ^14^ ^,^ ^15^ ^,^ ^17^ ^,^ ^18^ Previous findings showed that patients who initiated oseltamivir treatment with ≤ two days of symptom onset had a viral clearance within six days post drug treatment, one day earlier than the observed for patients who started on oseltamivir with > two days of symptom onset.^19^

The Brazilian Ministry of Health recommends a five-day treatment with oseltamivir for patients diagnosed with influenza.^20^ In the present study, we showed that only 10% of the patients receiving this treatment had an undetectable VL value within this five-day recommendation. In addition, within 10 days of treatment only 40% (4/10) of patients had a clearance of viral detection.

A study conducted with H1N1pdm09 positive patients hospitalised for severe pneumonia also showed a slower viral clearance regardless of oseltamivir treatment.^18^ Likewise, Meschi et al.^21^ observed that patients with H1N1pdm09 infections that presented severe clinical conditions had significantly prolonged viral shedding period. They also showed that treatment with antiviral drugs would reduce VL in the early stages of drug administration, but would not interfere as much when concerning viral shedding duration. Risk factors such as immunosuppression and the need for mechanical ventilation can also contribute for a longer viral shedding.^22^ The longest viral shedding described here was observed in patients reporting severe asthma under mechanical ventilation (patient 2) and lymphoma (patient 4).

The retrospective design was a limitation of the study, as patients were presented to physicians with a variable time of onset symptoms, which may have contributed to interfere with the final VL analysis. The small sample size for both the asymptomatic patients group and treated patients may also not be an ideal representative of the circulating viruses real proportions among those patients.

In conclusion, regarding the onset of symptoms, clinical manifestation, and age, H1N1pdm09 VL was only associated with patient symptoms (AS vs. symptomatic). In contrast, H1N1pdm09 infection rates does follow an age pattern.

Furthermore, it is important to assess the true contribution of asymptomatic patients on H1N1pdm09 viral transmission and infection, once, as shown here, they still presented a detectable viral shedding, even though at lower rates than those of symptomatic patients.

Finally, our results suggest that patients with underlying diseases and severe clinical symptoms may be considered for a prolonged antiviral treatment for a complete H1N1pdm09 viral clearance.

## References

[1] Iuliano AD, Roguski KM, Chang HH, Muscatello DJ, Palekar R, Tempia S (2018). Estimates of global seasonal influenza-associated respiratory mortality a modelling study. Lancet.

[2] World Health Organization (2019). Recommended composition of influenza virus vaccines for use in the 2020 Southern Hemisphere influenza season. https://www.who.int/influenza/vaccines/virus/recommendations/2020_south/en/..

[3] World Health Organization (2014). Influenza virus infections in humans (February 2014). homepage on the Internet.

[4] Ministério da Saúde (2018). Informe técnico: 20ª Campanha Nacional de Vacinação contra a Influenza. homepage on the Internet.

[5] Centers for Disease Control and Prevention (2019). Flu symptoms & complications. homepage on the Internet.

[6] Ip DK, Lau LL, Leung NH, Fang VJ, Chan KH, Chu DK (2017). Viral shedding and transmission potential of asymptomatic and paucisymptomatic influenza virus infections in the community. Clin Infect Dis.

[7] Launes C, Garcia-Garcia JJ, Jordan I, Selva L, Rello J, Munoz-Almagro C (2012). Viral load at diagnosis and influenza A H1N1 (2009) disease severity in children. Influenza Other Respir Viruses.

[8] Noh JY, Song JY, Hwang SY, Choi WS, Heo JY, Cheong HJ (2014). Viral load dynamics in adult patients with A(H1N1)pdm09 influenza. Epidemiol Infect.

[9] Lee N, Chan PK, Hui DS, Rainer TH, Wong E, Choi KW (2009). Viral loads and duration of viral shedding in adult patients hospitalized with influenza. J Infect Dis.

[10] US Food and Drug Administration (2018). FDA approves new drug to treat influenza. homepage on the Internet.

[11] Ministério da Saúde (2010). Protocolo para o enfrentamento à pandemia de influenza pandêmica (H1n1) 2009: ações da atenção primária à saúde. http://bvsms.saude.gov.br/bvs/publicacoes/protocolo_enfrentamento_influenza_2009.pdf..

[12] World Health Organization (2012). Interim global surveillance standards for influenza. homepage on the Internet.

[13] Duchamp MB, Casalegno JS, Gillet Y, Frobert E, Bernard E, Escuret V (2010). Pandemic A(H1N1)2009 influenza virus detection by real time RT-PCR is viral quantification useful?. Clin Microbiol Infect.

[14] Li CC, Wang L, Eng HL, You HL, Chang LS, Tang KS (2010). Correlation of pandemic (H1N1) 2009 viral load with disease severity and prolonged viral shedding in children. Emerg Infect Dis.

[15] Gorini da Veiga AB.Kretzmann NA.Correa LT.Goshiyama AM.Baccin T.Ardenghi P (2012). Viral load and epidemiological profile of patients infected by pandemic influenza A (H1N1) 2009 and seasonal influenza a virus in southern Brazil. J Med Virol.

[16] Loeb M, Singh PK, Fox J, Russell ML, Pabbaraju K, Zarra D (2012). Longitudinal study of influenza molecular viral shedding in Hutterite communities. J Infect Dis.

[17] Lau LL, Cowling BJ, Fang VJ, Chan KH, Lau EH, Lipsitch M (2010). Viral shedding and clinical illness in naturally acquired influenza virus infections. J Infect Dis.

[18] Lee N, Chan PK, Wong CK, Wong KT, Choi KW, Joynt GM (2011). Viral clearance and inflammatory response patterns in adults hospitalized for pandemic 2009 influenza A(H1N1) virus pneumonia. Antivir Ther.

[19] Li IW, Hung IF, To KK, Chan KH, Wong SS, Chan JF (2010). The natural viral load profile of patients with pandemic 2009 influenza A(H1N1) and the effect of oseltamivir treatment. Chest.

[20] Ministério da Saúde (2017). Protocolo de tratamento de influenza: 2017. homepage on the Internet.

[21] Meschi S, Selleri M, Lalle E, Bordi L, Valli MB, Ferraro F (2011). Duration of viral shedding in hospitalized patients infected with pandemic H1N1. BMC Infect Dis.

[22] Giannella M, Alonso M, Garcia de Viedma D.Lopez Roa P.Catalan P.Padilla B (2011). Prolonged viral shedding in pandemic influenza A(H1N1) clinical significance and viral load analysis in hospitalized patients. Clin Microbiol Infect.

