# Supplementary material for: Influenza A(H1N1)pdm09 infection and viral load analysis in patients with different clinical presentations
Source: Mem Inst Oswaldo Cruz. 2020 May 18;115:e200009. doi: 10.1590/0074-02760200009 (PMC7233266; doi:10.1590/0074-02760200009)
Supplement: Supplementary file 1 [file 1678-8060-mioc-115-e200009-s.pdf]

TABLE

Normalised (I) and non-normalised (II) data of viral loads (VL) from different patient groups (AS: asymptomatic; HP: hospitalised patients; and OP: outpatients), expressed in Log_{10} RNA copies/mL

	AS		HP adults		HP children		OP adults		OP children	
N	I	II	I	II	I	II	I	II	I	II
1	7.65	7.41	7.46	6.12	5.44	4.40	5.06	6.33	3.74	3.69
2	4.88	4.22	7.24	7.03	6.16	5.56	4.75	5.02	7.69	7.16
3	8.31	7.68	5.39	6.57	5.58	4.99	5.78	5.75	7.17	6.13
4	3.71	3.97	8.41	8.89	6.12	6.78	5.79	6.04	5.42	6.07
5	6.37	6.02	6.28	6.08	6.97	7.42	4.80	3.36	7.21	7.53
6	4.18	2.65	7.55	6.91	5.94	5.76	7.12	6.06	7.35	7.56
7	6.16	6.15	4.46	4.18	5.75	5.59	6.84	6.20	8.10	8.96
8	6.04	5.86	5.89	6.70	7.53	8.72	6.80	6.54	4.42	3.10
9	5.37	5.12	7.08	6.90	5.32	5.57	8.30	8.83	8.86	8.73
10	4.44	2.69	6.69	7.46	5.90	5.21	5.49	4.23	4.44	5.19
11	3.96	3.53	4.29	4.72	5.42	6.26	9.51	9.64	8.53	9.28
12	4.86	2.97	7.45	7.80	5.91	6.58	-	-	6.93	8.44
13	4.77	4.16	6.51	5.82	8.19	8.82	-	-	7.13	5.83
14	3.91	2.32	7.21	6.73	7.79	9.05	-	-	6.41	8.05
15	3.95	3.00	7.68	7.85	7.63	8.44	-	-	7.52	8.35
16	-	-	8.31	7.85	8.29	9.06	-	-	6.86	5.83
17	-	-	5.95	8.19	6.39	4.94	-	-	6.48	6.04
18	-	-	8.68	8.52	6.63	7.33	-	-	4.53	5.47
19	-	-	5.74	5.65	7.83	7.31	-	-	8.81	9.09
20	-	-	6.02	6.56	7.50	6.71	-	-	4.45	2.80
21	-	-	4.74	5.75	5.23	6.42	-	-	7.66	8.51
22	-	-	6.03	6.54	-	-	-	-	6.58	7.41
23	-	-	5.55	3.88	-	-	-	-	8.05	8.56
24	-	-	7.40	7.44	-	-	-	-	6.98	6.35
25	-	-	7.56	6.40	-	-	-	-	7.55	8.60
26	-	-	8.11	7.88	-	-	-	-	4.58	2.52
27	-	-	7.70	7.80	-	-	-	-	5.99	6.75
28	-	-	5.82	6.20	-	-	-	-	8.02	8.46
29	-	-	6.48	6.35	-	-	-	-	6.50	5.92
30	-	-	4.58	5.30	-	-	-	-	5.76	6.00
31	-	-	7.11	6.57	-	-	-	-	7.37	7.75
32	-	-	8.50	8.59	-	-	-	-	5.83	5.79
33	-	-	5.01	5.17	-	-	-	-	4.02	4.47
34	-	-	7.21	7.66	-	-	-	-	9.74	10.79
35	-	-	6.62	8.42	-	-	-	-	6.64	7.48
36	-	-	5.99	5.63	-	-	-	-	7.39	8.00
37	-	-	8.24	8.71	-	-	-	-	5.51	5.00
38	-	-	7.60	7.54	-	-	-	-	8.68	9.40
39	-	-	9.51	9.46	-	-	-	-	4.90	4.60
40	-	-	9.52	9.91	-	-	-	-	5.09	4.85

CC BY

	AS		HP adults		HP children		OP adults		OP children	
N	I	II	I	II	I	II	I	II	I	II
41	-	-	8.12	7.64	-	-	-	-	4.87	6.79
42	-	-	6.02	7.44	-	-	-	-	8.32	8.03
43	-	-	4.94	5.86	-	-	-	-	7.63	8.43
44	-	-	7.69	7.81	-	-			6.68	6.53
45	-	-	9.55	10.49	-	-	-	-	3.58	4.68
46	-	-	7.64	7.17	-	-	-	-	5.30	5.38
47	-	-	5.01	5.00	-	-	-	-	7.12	7.26
48	-	-	8.58	8.70	-	-	-	-	7.94	7.93
49	-	-	8.68	8.78	-	-	-	-	3.86	3.68
50	-	-	5.15	4.58	-	-	-	-	3.63	3.18
51	-	-	5.49	5.26	-	-	-	-	6.02	6.10
52	-	-	9.73	9.56	-	-	-	-	5.73	3.97
53	-	-	4.92	4.00	-	-	-	-	5.79	3.31
54	-	-	5.32	3.68	-	-	-	-	7.28	6.77
55	-	-	5.28	5.30	-	-	-	-	8.60	9.45
56	-	-	-	-	-	-	-	-	5.26	6.08
57	-	-	-	-	-	-	-	-	6.28	6.55
58	-	-	-	-	-	-	-	-	5.45	5.41
59	-	-	-	-	-	-	-	-	3.97	4.98
60	-	-	-	-	-	-	-	-	6.97	6.34
Mean	5.24	4.52	6.83	6.89	6.55	6.71	6.39	6.18	6.42	6.52
SD	1.41	1.76	1.46	1.59	1.03	1.46	1.51	1.80	1.53	1.91

N: samples; I: normalised data; II: non-normalised data; SD: standard deviation.
